# Anti-CD19 CAR-T cell therapy for acquired hemophilia A

**DOI:** 10.1038/s41375-025-02554-1

**Published:** 2025-03-08

**Authors:** Christian R. Schultze-Florey, Felicitas R. Thol, Krasimira Aleksandrova, Kalin Stoyanov, Rodrigo Gutierrez Jauregui, Lubomir Arseniev, Jana Leise, Stephan Klöß, Florian H. Heidel, Andreas Tiede

**Affiliations:** 1 Hematology, Hemostasis, Oncology, and Stem Cell Transplantation, Hannover Medical School, Hannover, Germany; 2 Cellular Therapy Centre, Hannover Medical School, Hannover, Germany; 3 Institute of Immunology, Hannover Medical School, Hannover, Germany; 4 Leibniz Institute on Aging, Fritz-Lipmann-Institute, Jena, Germany

**Keywords:** Autoimmune diseases, Immunotherapy

Acquired hemophilia A (AHA) is a rare autoimmune disorder caused by development of antibodies against clotting factor VIII (FVIII). It is characterized by severe spontaneous bleeding and prolonged bleeding after injury, often life-threatening. The therapeutic approach includes hemostatic medication (bypassing agents and recombinant porcine FVIII) to stop acute bleeding, and immunosuppressive therapy (IST) to eradicate autoantibody formation and induce remission [1]. Recently, the bispecific antibody emicizumab was introduced to prevent bleeding until remission of AHA [2]. Approximately 60–80% of patients reach complete remission (CR) over a variable period of weeks to months with established IST regimens. However, resistance to IST can pose a significant clinical challenge, as bleeding symptoms often persist, leading to continued need for hemostatic medications, intractable breakthrough bleeding, and an increased risk of death.

Anti-CD19 chimeric antigen receptor (CAR)-T cell therapy has shown promise in treating autoimmune diseases (AID) such as systemic lupus erythematosus, myositis, systemic sclerosis [3], rheumatoid arthritis [4], multiple sclerosis [5], stiff-person syndrome [6] and myasthenia gravis [7]. Considering AHA is an antibody-driven AID, CD19-directed CAR-T cell therapy represents a potential therapeutic option.

Here, we report the case of an IST-resistant AHA patient with intractable bleeding who achieved CR after autologous anti-CD19 CAR-T cell therapy. CR of AHA was defined as recovery of FVIII activity to >50 IU/dl, no bleeding, undetectable inhibitor, and cessation of IST [1].

In June 2023, a 39-year-old male was referred to our institution from a community hospital in hemorrhagic shock due to massive iliopsoas hematoma (Fig. 1A). He was diagnosed with AHA, with an inhibitor level reaching 534 BU/ml (Fig. 1B). Initial IST with dexamethasone, cyclophosphamide, and rituximab did not induce remission, and the patient continued to experience bleeding episodes despite prophylactic emicizumab. IST was progressively intensified with mycophenolate mofetil, ciclosporin A, and daratumumab. Although the inhibitor level declined, it remained consistently detectable, and FVIII activity was severely reduced (Fig. 1B). Over 10 months, the patient suffered life-threatening bleeds, including subarachnoid and subdural hemorrhages and massive cervical hematoma causing airway compression that required prolonged intubation. Hemostatic management with porcine FVIII was unsuccessful due to cross-reacting antibodies, necessitating high doses of rFVIIa.

**Fig. 1:**
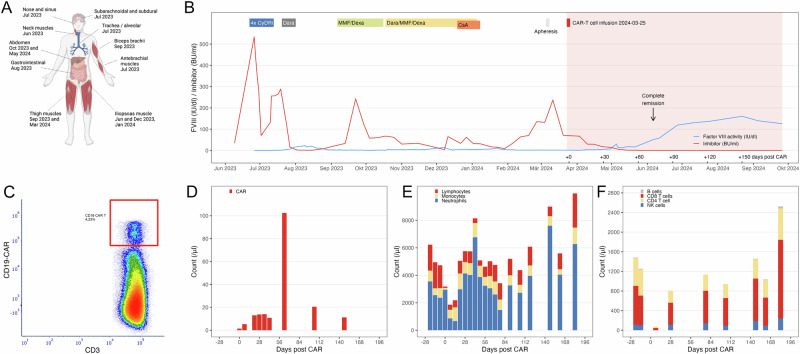
Impact of anti-CD19 CAR-T cells on acquired hemophilia. **A** Bleeding sites and time of occurrence. Location and time of bleeding event is shown. **B** Cause of FVIII activity and inhibitor during IST and CAR T-cell therapy. FVIII activity (blue line, in IU/dl or % of normal) was measured using a chromogenic activity assay. Anti-FVIII inhibitor (red line, in BU/ml) was measured using a modified Nijmegen-Bethesda assay. Abbreviations for IST regimens: CyDRi, cyclophosphamide, dexamethasone, rituximab; Dara, daratumumab; MMF/Dexa, mycophenolate mofetil, dexamethasone; CsA, ciclosporin A. **C** Detection of anti-CD19 CAR+ cells. Representative flow cytometric analysis of the percent of circulating anti-CD19 CAR+ cells of total T cells is shown. **D** Absolute count of CAR+ cells. Absolute count of CAR+ cells is shown over time. **E** Absolute counts of lymphocytes, monocytes and neutrophils. Absolute count per µl of lymphocytes, monocytes and neutrophils is shown over time. **F** Absolute counts of B cells, NK cells, CD4+ and CD8+ T cells. Absolute count of CD19+ B cells, CD4+ T cells, CD8+ T cells and CD16 + CD56 + NK cells per µl is shown over time. A was created in BioRender. Tiede, A. (2025) https://BioRender.com/i22q574. B and D-F were created with R version 4.3.0, the tidyverse package version 2.0.0.

Due to the refractory nature of this life-threatening condition, the patient received autologous anti-CD19 CAR-T cells. Autologous anti-CD19 CAR T-cells were manufactured at our institutional Cellular Therapy Center using a CliniMACS Prodigy® system (Miltenyi Biotec, Bergisch Gladbach, Germany) and a CD19 CAR lentiviral vector (Miltenyi Biotec, Bergisch Gladbach, Germany), as previously described [3]. Following lymphodepletion with fludarabine and cyclophosphamide as reported [3], we administered 1 × 10^6^ autologous anti-CD19 CAR T-cells per kg of body weight. IST was stopped prior to CAR-T infusion, while emicizumab was continued until CR was achieved.

The patient was monitored daily for symptoms of cytokine release syndrome (CRS) and immune effector cell-associated neurotoxicity syndrome (ICANS) [6]. The infusion was well tolerated, with no development CRS or ICANS.

Absolute counts of leukocytes, neutrophils and lymphocytes were determined with Sysmex XN1000 from peripheral blood. Absolute counts of peripheral blood leukocyte subsets were measured with BD Trucount tubes and the commercially available TBNK-assay (BD). CAR T-cells were determined with CD19 CAR Detection Reagent, and Biotin antibody (clone REA746; both Miltenyi Biotec, Bergisch-Gladbach, Germany). Flow cytometry was performed on FACS Lyric (BD) and Aurora spectral flow cytometer (Cytek) equipped with five lasers (355 nm, 405 nm, 488 nm, 561 nm, 640 nm) to acquire the primary data. Data analysis was performed using SpectroFlo version 3.3.0 (Cytek) and FCS Express™ 7 (Denovo).

The number of CAR + T cells in the peripheral blood showed a maximum increase by day(d) +64 followed by a continuous decline (Fig. 1C, D). Following lymphodepletion, we observed a drop of leukocytes with rapid recovery by d + 22. Importantly, ANC never dropped below 500/µl (Fig. 1E). ALC had recovered >1000/µl by day +63 (Fig. 1E). Re-occurrence of circulating B cells were detected at five months follow-up (Fig. 1F). Moreover, we observed a secondary hypogammaglobulinemia, which was treated with IVIG at IgG levels below 4 g/l.

The patient was evaluated for bleeding events and coagulation tests were performed at least weekly. FVIII activity was measured using a one-stage clot assay or a chromogenic assay with bovine components when the patient was receiving emicizumab. While the FVIII one-stage assay is artificially elevated in the presence of emicizumab, the bovine chromogenic assay is unaffected by emicizumab activity. Inhibitor levels were determined using a modified Nijmegen-Bethesda assay with chromogenic FVIII measurement and bovine components. Emicizumab activity was assessed using a diluted one-stage clot assay after heat inactivation of residual FVIII (50 °C, 30 min), as previously described [2].

On day (d)+41 post infusion, despite decreasing inhibitor levels the patient unexpectedly developed a hemorrhagic shock. CT angiography revealed an acute bleeding from the inferior mesenteric artery. Interventional radiologic exploration of the artery revealed a negative risk-benefit ratio for coiling. Under conservative treatment with rFVIIa for four days the bleeding was controlled. Thereafter, no further bleeding event occurred and no additional rFVIIa treatment was needed. As bleeding events are typically diffuse in AHA and the patient did not have any localized bleedings from an artery before during the course of his disease, we believe that the bleeding from the inferior mesenteric artery was rather coincidental than a sign of uncontrolled AHA. However, as the inhibitor was still detectable at this time point and the patient was not yet in remission, this bleeding event was considered as AHA-related.

The inhibitor level declined rapidly and became undetectable by d + 63 post-infusion. The last bleeding episode occurred on d + 41, while the inhibitor was still detectable, and no additional hemostatic treatment was needed thereafter. CR was achieved by d + 73, allowing discontinuation of emicizumab prophylaxis. At six months follow-up, the patient remained in stable CR. Regarding safety, no treatment-related toxicities were observed other than B cell aplasia and secondary hypogammaglobulinemia, and importantly, there were no infectious complications [8].

The CAR-T expansion dynamics of our AHA patient differs from previous reports in autoimmune diseases. Mueller et al. [3] described in their case series a peak expansion of circulating CAR-T cells after a median of 8.6 days post CAR-T infusion with a median number of 146 (IQR 61–697)/µl in the peripheral blood, while our patient showed only 5.3/µl CAR+ cells in the peripheral blood by day +7 post CAR-T infusion. Peak expansion occurred later, with the highest level of CAR+ cells detected on day +64 (102.5/µl), reaching a comparable peak expansion level as described. Of note, coinciding with this maximum number of circulating CAR+ cells the FVIII inhibitor became undetectable. Pharmacokinetic data from a CAR-T cell trial in solid tumors suggest the importance of antigen exposure for expansion and persistence of CAR+ cells [9]. While the circulating B-cells were rapidly deleted early post CAR-T infusion (Fig. 1F), the inhibitor was still detectable for the first two months post CAR-T. We therefore speculate that the late expansion CARs might be associated with detection of a dominant B-cell clone prior to this late time point, leading to an expansion of the CAR+ cell population. Future studies are needed to dissect potential differences in the pathophysiology of AHA in comparison to other autoimmune diseases such as SLE, iMM or SSc with regard to expression of the underlying B-cell clone.

This report demonstrates that anti-CD19 CAR-T cell therapy can induce remission in refractory AHA, representing a promising, safe, and potentially cost-effective treatment option. Further studies and prospective registries are recommended to more comprehensively assess the efficacy of CAR-T cell therapy in AHA.

## Data Availability

Data sharing requests of the primary data can be made available upon request via email with the corresponding author.
